# The effect of navigational expertise on wayfinding in new environments

**DOI:** 10.1016/j.jenvp.2010.03.003

**Published:** 2010-12

**Authors:** Katherine Woollett, Eleanor A. Maguire

**Affiliations:** Wellcome Trust Centre for Neuroimaging, Institute of Neurology, University College London, 12 Queen Square, London WC1N 3BG, United Kingdom

**Keywords:** Navigation, Taxi drivers, Spatial memory, Routes, Hippocampus

## Abstract

Becoming proficient at navigation in urban environments is something that we all aspire to. Here we asked whether being an expert at wayfinding in one environment has any effect on learning new spatial layouts. Licensed London taxi drivers are among the most proficient urban navigators, training for many years to find their way around a complex and irregularly-laid out city. We first tested how well they could learn the layout of an unfamiliar town compared with a group of non-taxi drivers. Second, we investigated how effectively taxi drivers could integrate a new district into their existing spatial representation of London. We found that taxi drivers were significantly better than control participants at executing routes through the new town, and representing it at a map-like survey level. However, the benefits of navigational expertise were not universal. Compared with their performance in the new town, taxi drivers were significantly poorer at learning the layout of a new area that had to be integrated with their existing knowledge of London. We consider reasons for this picture of facilitation and limitation, in particular drawing parallels with how knowledge acquisition occurs in the context of expertise in general.

## 1 Introduction

Finding your way around a large city can be challenging, all the more so if the layout of the city in question is complex (Lynch, 1960). Although cities in North America are often laid out in a regular grid pattern, in contrast many European cities have chaotic, unpredictable layouts, a prime example being London (UK). Despite its complexity, there is a group of people who are expert at navigation around central London. Licensed London taxi drivers undergo gruelling training, often lasting four years, before taking stringent examinations in order to obtain an operating licence. Taxi drivers must learn the layout of over 25,000 streets, thousands of places of interest, and be conversant with London’s numerous one-way traffic systems.

Previous studies have documented taxi drivers’ expertise in London knowledge and wayfinding compared with control participants (Woollett & Maguire, 2009; Woollett, Spiers, & Maguire, 2009), including London bus drivers (Maguire, Woollett, & Spiers, 2006), who also drive customers along London’s streets, but using a constrained set of routes. Notably, however, the wayfinding expertise of taxi drivers seems to come at a cost. They perform more poorly than control participants on several memory tests involving new visuo-spatial materials. Specifically, they recalled less details of a complex figure (Rey–Osterrieth complex figure; Osterrieth, 1944) after a delay (Maguire et al., 2006; Woollett & Maguire, 2009). Similarly, they took longer to learn associations between sixteen objects and locations on a table-top array, and also had poorer recall of the object–location pairs after a delay (Woollett & Maguire, 2009).

Thus, taxi drivers who excel at wayfinding around a complex city were poor at acquiring some types of new visuo-spatial information that control participants could learn with ease. However, it could be argued that the types of table-top tasks employed in previous studies did not directly assess the spatial processing that taxi drivers typically engage in when immersed in a large-scale complex space such as an urban environment. As such, a key question remains unanswered, namely, does being a very skilled navigator in one environment confer an advantage when learning the layout of a new and unfamiliar environment? Or, do the decrements observed on some table-top spatial learning and recall tasks in taxi drivers suggest that the ability to learn a new spatial layout might be compromised in the context of navigational expertise? Both outcomes have implications for understanding potential mechanisms of wayfinding, and how environmental knowledge is represented. For instance, if wayfinding expertise facilitates learning a new environment, this could suggest that expertise might in part be underpinned by the development and use of generalisable and effective strategies for wayfinding. By contrast, if learning a new environment is constrained, this may indicate that expertise and detailed existing environmental knowledge limits the capacity for processing or storage of new spatial layouts, or results in a bias towards existing knowledge, similar to some models of ageing (Wilson, Gallagher, Eichenbaum, & Tanila, 2006).

The central aim, therefore, of this study was to ascertain if taxi drivers’ navigational expertise in London affected their ability to learn the layout of a new environment. A range of tests used in previous wayfinding studies (e.g. Beck & Wood, 1976; Blades, 1990; Gale, Golledge, Pellegrino, & Doherty, 1990; Golledge, Smith, Pellegrino, Doherty, & Marshall, 1985; Kirasic & Mathes, 1990; Maguire, Burke, Phillips, & Staunton, 1996; Rosenbaum, Winocur, Grady, Ziegler, & Moscovitch, 2007; Rovine & Weisman, 1989; Woollett & Maguire, 2009) was employed to assess knowledge of environmental features and topographical details, spatial relationships between landmarks, planning and execution of routes, and whether an overall survey-like representation had been acquired (Golledge, 1999; Montello, 1998; Siegel & White, 1975). We examined the acquisition of new environmental knowledge in two ways; first, by testing how well taxi drivers could learn the layout of a previously unfamiliar town compared with a control group of non-taxi drivers. Second, we investigated how effectively taxi drivers could integrate a new district into their existing spatial representation of London. In this latter case, it was not possible to test control participants, as they simply did not have a comparable knowledge of London’s layout as a basis for integration. Consequently, the key comparison here was between taxi drivers’ performance in the entirely new town and their performance in the new area of London.

## 2 Materials and methods

### 2.1 Participants

Thirty-eight healthy male volunteers participated in the study. Of these, 20 were licensed London taxi drivers, and 18 were control participants. All participants gave informed written consent to participation in the study in accordance with the local research ethics committee. The background details of the two groups are shown on Table 1. All taxi drivers had completed “The Knowledge” training, had passed the necessary Public Carriage Office examinations, and obtained a full (green badge) licence. Taxi drivers had on average 13.27 years of taxi driving experience (SD 7.86; range 0.5–25 years). All control participants were resident in greater London. None of the control participants had worked as licensed London taxi drivers or mini-cab drivers. None was training to be a licensed taxi driver or had ever been involved in such training. Taxi drivers and control participants did not differ in terms of age (*t*(36) = 1.85; *p* = 0.07), handedness (Oldfield, 1971) (*t*(36) = 0.24; *p* = 0.8), or age when they left school (*t*(36) = 0.7; *p* = 0.4). Visual information processing and abstract reasoning skills were assessed using the Matrix Reasoning sub-test of the Wechsler Abbreviated Scale of Intelligence (Wechsler, 1999). The mean scaled score for both groups did not differ significantly (*t*(36) = 0.7; *p* = 0.4). An estimate of verbal IQ was obtained using the Wechsler Test of Adult Reading (Wechsler, 2001). Data for two taxi drivers and two control participants were not obtained – although they were very proficient in English, it was not their first language, a requirement of the test. Verbal IQ estimates for both groups were in the average range, and did not differ significantly (*t*(32) = 1.05; *p* = 0.3).

### 2.2 Environments and tests

The effect of navigational expertise on environmental learning and knowledge was assessed in two ways, first using an unfamiliar town – “New Town”, and second using modifications to the familiar environment of London (UK) – “London”.

#### 2.2.1 New Town

**Fig. 1:**
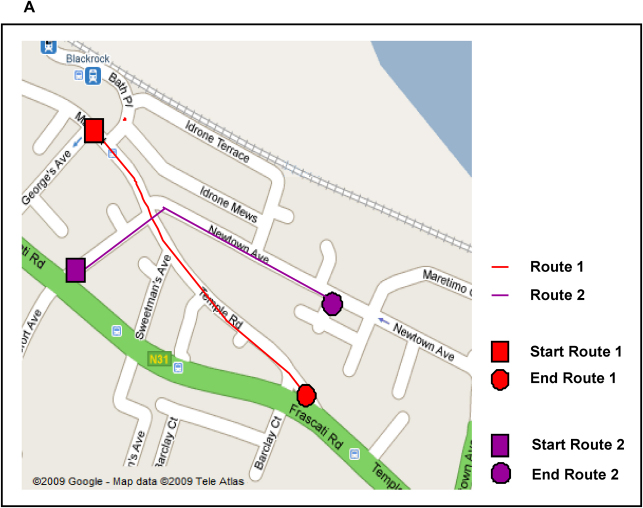
Map of New Town. The two overlapping routes are shown. Note that participants never saw this map. Map © Google Maps.

Film footage was acquired of navigation along two routes through an urban environment (see Fig. 1). Stimulus material was adapted from that used by (Maguire et al., 1996), and featured a town called Blackrock which is south of Dublin City, Ireland. There was no footage in common between the two routes except for brief navigation across one point of overlap at a central road junction. Footage was shot in colour with a wide angle lens, at eye-level, and at average walking pace. The camera panned from side to side, to simulate natural viewing and in order to include the salient features along each route such as prominent buildings and shop fronts. When a road junction was reached, the pace slowed and the camera panned down all the elements of the road junction before moving on. The two routes were presented one after another on a computer screen, with a total viewing time of 4 min 53 s. As the primary domain of interest in this study was visual, sound was not included. None of the participants was familiar with the environment. The film was shot in the main shopping area of the town. Landmarks were defined to participants as prominent buildings and distinctive shops/businesses. The following instructions were also given at the start of the test:

You are going to see films of navigation along two overlapping routes through a town. It will proceed at a brisk walking pace and the camera will pan and move as if you’re looking around while walking along. You should try to remember as many of the landmarks (buildings, shops, etc) as possible. Ignore cars, buses and people as they are not important. After each viewing of the footage, you will see a series of very short film clips – you will need to indicate if they were part of the routes you have just seen or not.

Participants viewed the footage four times. To ensure they paid attention and to check that learning was occurring, after each viewing, participants were shown four clips lasting 3 s each and asked to indicate if they formed part of the route they had just seen or not. Two were actual clips from the routes, and two were similar but never-seen distractor clips. There were new clips for each viewing (i.e. each test clip was shown only once during learning).

After participants had viewed the footage four times, they then completed a number of tests designed to assess their knowledge of the environment they had just learned.

##### 2.2.1.1 New Town scene recognition memory test

Recognition memory for environmental features and topographical details was assessed by showing participants 32 colour photographs of scenes. Twelve were scenes from route 1 in New Town, twelve were scenes from New Town’s route 2, and eight were distractor scenes that were not from New Town, but were visually similar. Target and distractor scenes were randomly intermixed. The format was a yes/no recognition memory test where participants were asked to state whether they recognised each scene as a part of New Town or not. The test was not formally timed, however subjects took on average 2–5 s per photograph.

##### 2.2.1.2 New Town proximity judgements

**Fig. 2:**
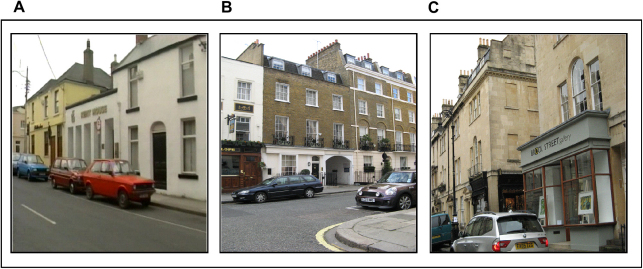
Example views from the three environments. A. Photograph taken in New Town. B. A view from existing London. C. A photograph from new London.

Participants’ knowledge of the spatial relationships between landmarks in New Town was tested using a proximity judgements task. Stimuli were colour photographs each depicting a New Town landmark (see example in Fig. 2A). On each trial, subjects had to judge which of two other New Town landmarks was closer (as the crow flies) to the target landmark. There were ten trials of which three trials comprised landmarks solely within route 1, three trials where landmarks were all within route 2, and four trials where landmarks were taken from both routes. The test was not formally timed, however participants took on average took 5–8 s per trial.

##### 2.2.1.3 New Town route execution

To test the ability to plan and ‘navigate’ along routes, participants were given a photograph of a New Town landmark labelled ‘start point’ and another labelled ‘end point’. Six additional landmark photographs were then supplied, with the instruction to place these in the correct order that they would be passed en route between the start and end points. There were four route execution trials, one where all landmarks were in route 1, another where all landmarks were in route 2, and two that spanned both routes (i.e. started in one route and ended in the other). Each correctly placed photograph was given a score of 1. The mismatch between the presented and correct order was derived by calculating the vector distance [Σ(*x* − *y*)^2^] between the position presented for each photograph (*y*) and what the correct position for that photograph should have been (*x*). Thus a score of zero indicates a perfect match between the presented and actual placements for a given trial.

A previous study found no performance differences when within and between route trials were compared on either the proximity judgements task or the route execution task (Maguire et al., 1996). Consequently, in this study, analysis was collapsed across within and between trial types.

##### 2.2.1.4 New Town sketch map

Participants were asked to draw a sketch map of the routes seen in the film footage, including any landmarks they could recall. It was made clear that drawing ability would not be assessed. Standard A3 size white paper (297 × 420 mm/11.7 × 16.5 inches) and pencils were used. Participants were not provided with erasers. If they wished to restart, they were permitted to do so on the reverse of the sheet, although this never occurred. In two cases, more paper was requested as the map exceeded the space on one page. The following variables were examined:

• number of road segments, where a segment referred to a section of road between road junctions
• number of road junctions
• number of correct landmarks
• landmark placement, with a maximum of three points per landmark, where one point was given if the landmark was on the correct side of the road, one point if it was correctly placed in relation to nearby road junctions, and one point if it was in the correct sequence relative to preceding and subsequent landmarks
• orientation score – an experimenter rating that assessed how the roads and layout were orientated, on a scale of 1–5, where 1 was poor and incorrect…5 was good and accurate orientation
• overall map categorisation score – an experimenter rating on a scale of 1–6. The map categorisations for ‘New Town’ were based on the range of previous data obtained using the same stimuli (Maguire et al., 1996). As such, the categories represented distinct progressions in the quality of map coherence.
1. The two routes were merged into one
2. There were two routes, but they were perceived as separate
3. The two routes were close together, but not joined accurately
4. Some elements across the two routes were joined up, but integration was broadly lacking
5. The two routes were integrated, but there were some inaccuracies in layout
6. Correct integration across the two routes, survey-like map, accurate and easy to follow

Twenty percent of the sketch maps were also scored by a second rater, blind to group membership; the inter-rater correlation was 0.99.

#### 2.2.2 London

In the first instance we sought to verify if taxi drivers had significantly more knowledge about London’s layout than the control participants. To do this we used a test shown in several previous studies to be a reliable measure of topographical knowledge (Maguire et al., 2006; Woollett & Maguire, 2009). The London landmarks proximity judgements test comprised colour photographs each depicting a famous London landmark. On each trial, subjects had to judge which of two other London landmarks was closer (as the crow flies) to the target London landmark. There were 10 trials. The test was not formally timed, however subjects on average took 5–8 s per trial.

Having assessed how well taxi drivers could learn a novel environment (see New Town above), we were also interested in whether they could integrate new environmental knowledge into their existing cognitive representation of London. To examine this, we devised the London test. The London test was only given to taxi drivers, as control participants could not be compared to taxi drivers in their basic level of London knowledge.

**Fig. 3:**
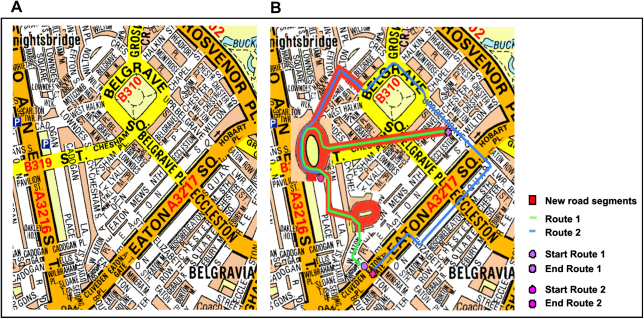
Map of London. A. London as it is normally. B. A map showing existing London integrated with ‘new’ London, where modifications are depicted in red. Note that participants never saw these maps. Maps reproduced by permission of Geographers’ A–Z Map Co. Ltd. © Crown Copyright 2005. All rights reserved. Licence number 100017302.

Footage was presented on a computer screen and comprised continuously updating colour photographs; each photograph was on the screen for 2 s before the next photograph in the sequence appeared. Pilot testing determined this was a comfortable pace for viewing. The photographs (see Fig. 2B) were taken at eye level, with a wide angle, and in an evenly-spaced fashion to mimic walking, giving the impression of navigation along routes. Participants (and piloting) confirmed that this readily conveyed the sensation of walking through the environment. The routes depicted in the photographs were made up of an existing part of London with some modifications. Modifications involved diverting participants from existing London into new areas they were not familiar with – ‘new London’ (see Fig. 3). Photographs depicting these new areas were shot in another city (Bath, UK) with buildings of broadly similar character and historical period as the existing part of London (see Fig. 2C). None of the participants was familiar with this other city. Several criteria guided the development of the London task: first, we needed to match the overall appearance and architecture of that part of existing London with somewhere similar (but not too distinctive in and of itself). Next, we did not want to have too many links between existing and new London, but rather a sufficient number to test the taxi drivers whilst preserving the logic of connections between existing and new, without too much confusion or a sense of ‘weirdness’. Finally, we wanted to approximately equate the amount of information in the London task with that in New Town.

As with the New Town task, there were two overlapping routes. Each route was made up of segments from existing London and new London. Overall, the London test comprised 47% existing London and 53% new additions. We elected to use photographic stimuli in this instance, rather than film as used for the New Town task, because this enabled a seamless transition between existing and new London. All transitions between existing and new London involved a turn (left or right) in order to avoid transition points appearing unrealistic or confusing. Text instructions appeared on the screen indicating that a turn was coming up. The difference between the film and photographic presentations was never raised as an issue by participants or during piloting. Previous work has suggested a relatively high correspondence in response to colour photographs and on-site presentations (Brush, 1979; Craik & Feimer, 1987; Daniel & Boster, 1976). Whilst we cannot rule out a potential effect of presentation mode, given the naturalistic feel associated with both types of stimuli, we do not believe it significantly affected the results. The following instructions were given at the start of the London test:

You are going to see footage of navigation along two overlapping routes through London. The area will seem familiar but some of it has been rebuilt after a large number of buildings collapsed, and some of the roads have also been replaced. It will proceed at walking pace and the camera will pan and move as if you’re looking around while walking along. You should try to remember as many of the landmarks (buildings, shops, etc) as possible. Ignore cars, buses and people as they are not important. After each viewing of the footage you will see a series of very short film clips – you will need to indicate if they were part of the routes you have just seen or not.

Learning and memory was assessed in the same way as described above for New Town. There were four exposures to the footage with four short test clips after each viewing, one from existing and one from new London, plus two distractor clips. There were new clips for each viewing (i.e. each clip was shown only once during learning). Testing then proceeded with a scene recognition memory test, a proximity judgements test, a route execution test, and the drawing of a sketch map. For the London test, three additional adjustments were made. (1) The London scene recognition test, just like the New Town test, comprised twelve scenes from each route and eight distractor scenes. Ten of the target scenes were from the new additions to London, while fourteen scenes were from existing London. (2) The sketch maps were scored in two ways. Initially the complete sketch maps were analysed in an identical fashion to New Town. In order to examine the taxi drivers’ knowledge of specifically the new additions to London in the context of existing knowledge, the aspects of the map relating to existing and new London were also scored separately. (3) An additional sketch map experimenter rating was included for the London test in order to capture how well the existing and new parts of London were integrated, on a scale of 1–5, where 1 = little or no integration… 5 = good integration. Twenty five percent of the New London sketch maps and experimenter ratings were also scored by a second rater; the inter-rater correlation was 0.99.

### 2.3 Procedure

Each participant was tested individually. The New Town and London tests were administered to the taxi drivers in separate sessions that were at least 1 week and no more than 3 weeks apart. Participants were debriefed following the two sessions in order to ascertain feedback on how they found the tests, and to make comparisons between the two learning experiences.

### 2.4 Data analysis

Group comparisons relating to participant characteristics were made using two-tailed *t*-tests. For the main between-group analyses, data were screened for outliers, homogeneity of variance, and to ascertain if the data were normally distributed. Multivariate analysis of variance (MANOVA – Hotelling’s trace multivariate test) was employed using the general linear model with the significance threshold set at *p* < 0.05. Group (taxi drivers, control participants) was the independent variable, and the main New Town environmental knowledge measures were the dependent variables. Where MANOVA indicated a significant effect, the between-participant tests were employed to ascertain the source of the significance with a threshold of *p* < 0.05. For the main within-group analyses (taxi drivers only), data were screened for outliers, homogeneity of variance, and to ascertain if the data were normally distributed. One-way analysis of variance (ANOVA) was employed with the significance threshold set at *p* < 0.05. Two separate one-way ANOVAs were performed. In the first, town (New Town, London) was the independent variable, and the main environmental knowledge measures were the dependent variables. In the second ANOVA, environment type (New Town, existing London, new London) was the independent variable, and a range of the environmental knowledge measures were the dependent variables. Where ANOVA indicated a significant effect, post-hoc tests using Bonferroni correction were employed to ascertain the source of the significance with a threshold of *p* < 0.05. In addition, where variables were not included in the ANOVAs, two-tailed paired *t*-tests were employed (see further details in Results). To reduce the chance of Type I error, Bonferroni correction (*p* = 0.05/*n*, where *n* is the number of *t*-tests) was applied. Effect size was calculated using Cohen’s *d*, and is reported where relevant (i.e. for significant differences between two means). Correlations were also performed between the number of years taxi driving and all of the environmental knowledge measures, although none were significant.

## 3 Results

### 3.1 New Town

**Fig. 4:**
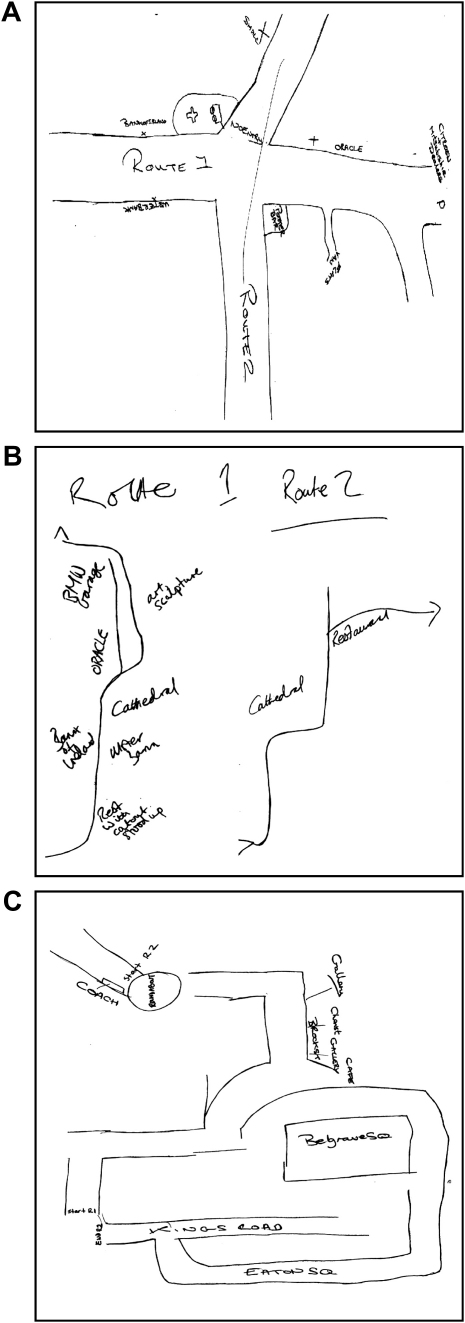
Example sketch maps. A. A taxi driver’s sketch map of New Town. B. A control participant’s sketch map of New Town. C. A map of existing and new London, as drawn by the same taxi driver whose map of New Town is shown in A.

Mean test scores are shown on Table 2. There was no difference between taxi drivers and control participants in their ability to recognise the film clips that were shown during the initial viewing phase (*t*(36) = 0.88; *p* = 0.38). A MANOVA was then performed with Group (taxi drivers, control participants) as the independent variable, and the main New Town environmental knowledge measures (scene recognition, proximity judgements, route execution, sketch map number of road segments, sketch map number of road junctions, sketch map number of landmarks, sketch map landmark placement) as the dependent variables. This revealed a significant difference between the groups (*F*(1,30) = 2.52; *p* = 0.03). The source of this difference was investigated using the tests of between-participant effects produced by MANOVA. There were five main effects. Taxi drivers were significantly better than control participants on route execution (*F*(1,36) = 5.72; *p* = 0.02; *d* = 0.77), on sketch map number of road segments (*F*(1,36) = 13.96; *p* = 0.001; *d* = 1.22), sketch map number of road junctions (*F*(1,36) = 8.64; *p* = 0.006; *d* = 0.95), sketch map number of landmarks (*F*(1,36) = 6.11; *p* = 0.01; *d* = 0.82), and sketch map landmark placement (*F*(1,36) = 5.60; *p* = 0.02; *d* = 0.77). In addition, taxi drivers were also rated better on the sketch map orientation score (*t*(36) = 2.52; *p* = 0.01; *d* = 0.82) and on the overall sketch map categorisation score (*t*(36) = 3.27; *p* = 0.002; *d* = 1.07) (see Fig. 4 for example sketch maps).

### 3.2 London

We first sought to examine whether taxi drivers had significantly better knowledge about London’s layout than the control participants; this was measured using the London landmarks proximity judgements test. Taxi drivers mean score on this test was 8.75/10 (SD 0.91) compared with 7.61 (1.19) for control participants, with taxi drivers performing significantly better (*t*(36) = 3.32; *p* = 0.02; *d* = 1.08).

We then compared taxi drivers’ performance on the London test with their performance in New Town (see mean London scores on Table 3). The raw scores were converted to proportion scores to make comparison possible across the two towns.

There was no difference in the ability to recognise the film clips that were shown during the initial viewing phase of New Town and London (*t*(19) = 0.32; *p* = 0.74). A one-way ANOVA was used to test for differences between London and New Town on seven environmental knowledge measures (scene recognition, proximity judgements, route execution, sketch map number of road segments, sketch map number of road junctions, sketch map number of landmarks, sketch map landmark placement). There were two main effects. Scene recognition was better for London compared with New Town (*F*(1,38) = 31.29; *p* = 0.001; *d* = 1.77), while sketch map number of landmarks was better for New Town (*F*(1,38) = 7.25; *p* = 0.01; *d* = 0.85). There was no effect for proximity judgements (*F*(1,38) = 0.11; *p* = 0.73), sketch map number of road segments (*F*(1,38) = 0.15; *p* = 0.69), sketch map number of road junctions (*F*(1,38) = 2.34; *p* = 0.13), sketch map number of correctly placed landmarks (*F*(1,38) = 2.38; *p* = 0.13), or route execution (*F*(1,38) = 3.23; *p* = 0.08). Experimenter ratings of sketch map orientation (*t*(19) = 1.63; *p* = 0.12) and overall sketch map categorisation (*t*(19) = 0.96; *p* = 0.35) did not differ between the two environments.

Our main interest was in examining specifically the acquisition of new environmental knowledge on its own (New Town), and in the context of existing knowledge (new additions to London - see Table 4 for data relating to existing and new London separately). A one-way ANOVA was used to test for differences among three environment types, New Town (NT) existing London (EL) and new London (NL) on five environmental knowledge measures (scene recognition, sketch map number of road segments, sketch map number of road junctions, sketch map number of landmarks, sketch map landmark placement sketch map scores). It was not possible in this instance to include sufficient trials to compare New Town and new London on the proximity and route execution tests. There were four main effects across the three environment types, scene recognition (*F*(2,57) = 26.18; *p* = 0.001), sketch map number of road segments (*F*(2,57) = 4.32; *p* = 0.001), sketch map number of road junctions (*F*(2,57) = 8.22; *p* = 0.001), and sketch map number of landmarks (*F*(2, 57) = 4.77; *p* = 0.012). There was no effect for number of correctly placed landmarks (*F*(2,57) = 2.35; *p* = 0.105).

Bonferroni post-hoc comparisons of the three environment types showed that the number of correctly recognised scenes in EL was significantly higher than the number of correctly recognised scenes in NL (*p* = 0.001; *d* = 1.65), and in NT (*p* = 0.001; *d* = 2.50). There was no difference in the number of correctly recognised scenes between NL and NT (*p* = 0.25).

The number of road segments recalled for EL was significantly higher than the number of road segments recalled for NL (*p* = 0.001; *d* = 1.64), while the number of road segments recalled for NT was significantly higher than the number of road segments recalled for NL (*p* = 0.004; *d* = 1.08). There was no difference in the number of road segments recalled for EL and NT (*p* = 0.198).

The number of road junctions recalled for EL was significantly higher than the number of road junctions recalled for NL (*p* = 0.002; *d* = 1.12), while the number of road junctions recalled for NT was significantly higher than the number of road junctions recalled for NL (*p* = 0.004; *d* = 0.98). There was no difference in the number of road junctions recalled for EL and NT (*p* = 1.00).

The number of landmarks recalled for NT was significantly higher than the number of landmarks recalled for NL (*p* = 0.009; *d* = 1.11). There was no difference in the number of landmarks recalled for EL and NT (*p* = 0.28), or for EL and NL (*p* = 0.51).

Experimenter ratings of sketch map orientation (*t*(19) = 8.54; *p* = 0.001; *d* = 2.28) and overall sketch map categorisation (*t*(19) = 6.96; *p* = 0.001; *d* = 1.65) were significantly better for New Town compared to new London.

Considering integration between the existing knowledge of London’s layout and the new environmental information, several other scores are relevant. There were three major integration points (road junctions) between existing and new London. On average, taxi drivers included 1 (SD 0.77) on their sketch maps. The London sketch maps were also scored by the two raters for integration on a scale of 1–5, where 1 = little or no integration… 5 = good integration. The average integration score was 2.6 (SD 1.14). In a debriefing session taxi drivers were also asked how they found doing the London task relative to New Town. The overwhelming response was that they found the London task more difficult than learning New Town. Some of the reasons given included: “*I couldn’t link the old with the new*”; “*It would have been easier if I had to learn just the new London bits. It was confusing to add them to the old London*”; “*Very hard to remember the new parts*”; “*It was confusing to put the new parts into London*”; “*I found it a lot harder than New Town. I found it hard to integrate the new parts into London*”; “*I couldn’t link the new parts into London properly*”.

Note the results reported here pertain to male participants. Future studies of female participants will be needed in order to assess the generalisability of these findings across genders.

## 4 Discussion

In this study, we examined whether being a very skilled navigator in one environment had any effect on learning the layout of an entirely new environment. We found that it did, with licensed London taxi drivers significantly better than control participants at executing routes through a new town, and representing this environment at an overall map-like survey level. However, the benefits of navigational expertise were not universal. Compared with their performance in the new town, taxi drivers were significantly worse when tested on their knowledge of the layout of a new area that had to be integrated into their existing representation of London. We discuss each of these results in turn.

Both control participants and taxi drivers learned New Town at the same rate, were comparable in their ability to recognise scenes from New Town from among similar-looking distractors, and were able to make proximity judgements between landmarks to an equivalent level. Thus, the significantly better performance by taxi drivers in executing routes through New Town and across all the sketch map variables was not attributable to differences in basic topographical knowledge. Rather the advantage for taxi drivers was in being able to plan and execute routes, and in possessing a survey-like representation of the town as exemplified in sketch maps. Examination of the representative sketch map in Fig. 4A, illustrates that taxi drivers appreciated how the two routes overlapped, and were able to integrate them into a reasonably coherent spatial map of the area. By contrast, the control participants, whilst acquiring knowledge of the two routes separately, were generally less able to make the discrete representations cohere (Fig. 4B).

Overall, this finding suggests that wayfinding expertise in a specific environment is not only a matter of accruing a large amount of information about the layout and content of that environment. The fact that experts are benefitted when learning a new environment means that aspects of wayfinding expertise generalise. What might these aspects be? Taxi drivers undergo years of training where they have to pay close attention to multiple complex routes as well as salient landmarks, and learn how the routes across a large city relate to each other. Similarly in their job, day in day out, they are required to plan and execute routes (Spiers & Maguire, 2008). Clearly these general attentional, learning and memory mechanisms are finely-tuned and readily called upon when they are required to learn a new town. In the current task, and in line with their training and experience, it is likely that they paid more attention than control participants to the content of the two routes, when salient landmarks appeared and in what order, and in particular to how the routes fitted together.

While taxi drivers are highly skilled at wayfinding around London, and were advantaged when learning a new environment, acquisition of novel spatial information in the context of an existing knowledge domain was significantly poorer in comparison. Thus the cognitive mechanisms that were at play in New Town were seemingly not in operation to the same degree in the new parts of London. For example, a number of the sketch map measures such as number of road segments, number of road junctions, overall orientation and overall map categorisation scores were significantly lower for new London suggesting the taxi drivers failed to integrate existing and new parts of London into a holistic representation. Further evidence for this lack of an overall survey-like representation was in the low ratings for integration between the two parts of London, the fact that taxi drivers on average only recalled one of the three major junctions between existing and new London, and most clearly of all in the direct comments of the taxi drivers in the debriefing. They unanimously found the London task much more challenging than learning a new town from scratch. Why might this be?

There may be parallels between our findings and those of previous research in the realm of expertise more generally. While it has been shown that experts’ performance generally surpasses that of novices in domains such as chess, bridge, sport, music, and physics (Bootsma & Van Wieringen, 1990; Charness, 1987; Chase & Simon, 1973; Ericsson, Krampe, & Tesch-Romer, 1993; Reif & Allen, 1992), such experts can be prone to making mistakes and be more errorful than novices in some circumstances (Hecht & Proffitt, 1995; Norman, 1981; Saariluoma, 1992; Wiley, 1998). Luchins (1942) was one of the first to demonstrate this experimentally by showing the Einstellung effect in a water jug experiment. Participants were trained to solve a series of problems using a fixed solution. When tested on new problems that were similar to the ones used during the training phase, the majority of participants failed because they applied the fixed solution instead of finding the most appropriate solution for a given trial. Luchins argued that experts’ knowledge can make them unable to adapt to new task demands thereby missing the optimum solution to a problem. This effect has also been found within specific domains of expertise, where experts were reported to show increased accuracy, speed and capacity compared to novices, but they made more errors when presented with new problems that closely resembled their knowledge base (Frensch & Sternberg, 1991; Woltz, Gardner, & Bell, 2000).

Ericsson (2003) suggested that after a period of training, performance becomes automated and experts may lose conscious control over the execution of skills, making intentional modifications difficult, resulting in errors within the domain in which they hold their expertise. This suggests that performance is negatively affected when there is increased similarity between old and new information, or where there are overlapping demands between old and new stimuli (Woltz et al., 2000). Memory in aged adults has also been shown to be negatively affected when there is greater similarity between contexts and objects (Henkel, Johnson, & De Leonardis, 1998; Parkin et al., 2001). We suggest that the Einstellung effect in experts can be regarded as a form of memory interference. Thus, when operating in a new environment which is distinct from the environment where they have their expertise, the finely-honed strategies of London taxi drivers offers them a distinct advantage. By contrast, when presented with new information to learn that is similar to their existing knowledge, their poorer performance may reflect expert inflexibility and an inability to inhibit access to existing (and now competing) memory representations.

When working in London, taxi drivers are required to incorporate new information into their representation of the city on a daily basis, such as new traffic flows and road works information. If they had difficulty with assimilating this new information then we might expect a large proportion of taxi drivers to have problems carrying out their job. But this is not the case; taxi drivers in London usually stay working successfully in the profession for decades. It is likely, however, that the changes to the environment that they encounter are incorporated gradually, with difficulties overcome by repeatedly travelling along the modified routes. In our task, they had only four exposures in which to acquire the novel information. Interestingly, in the debriefing session, a number of taxi drivers noted the parallel between the difficulties they had with our new London task, and a situation that arose a number of years ago with the development of a new area in London. When the Canary Wharf district was opened, taxi drivers had great difficulty getting to know the area, and said that it took a long time before they were able to navigate successfully there. It may be that our sudden introduction of radical changes caused the taxi drivers to stop using their normal survey representation and instead they reverted back to route-based strategies (Evans, Marrero, & Butler, 1981; Hart & Moore, 1973; McNamara, 1986; Siegel, 1981; Siegel & White, 1975). The latter may have been overburdened by the amount of change and the mismatch between expected, well-known sequences of intersections and the new experiences.

Previous studies of London taxi drivers documented their poor performance on table-top tests of visuo-spatial memory. Specifically, taxi drivers recalled less details of a complex figure (Rey–Osterrieth complex figure; Osterrieth, 1944) after a delay compared with control participants (Maguire et al., 2006; Woollett & Maguire, 2009). Similarly, they took longer to learn associations between sixteen objects and locations on a table-top array, and also had poorer recall of the object–location pairs after a delay (Woollett & Maguire, 2009). It is not immediately clear why deficits should be seen on these tests which involved exclusively new material, and in the current London task which involved the integration of new with existing knowledge in large-scale space. Future work will be required to try and understand the relationship between these sets of findings and to identify possible processes they might have in common that are compromised in the context of navigational expertise.

In conclusion, the main aim of this study was to assess whether wayfinding expertise in one urban environment had any effect on learning a new environment, and when incorporating novel information into an extant spatial representation. We have demonstrated that expertise is coupled with an advantage over novices for large-scale spatial layouts that are distinct from existing knowledge, whilst at the same time placing limits on experts’ performance within their specific domain of expertise.

## Figures and Tables

**Table 1:** Participant characteristics.

Measure	Taxi drivers Mean (SD)	Control participants Mean (SD)
Age (years)	42.1 (5.37)	38.72 (5.85)
Education (age left school, years)	16.45 (0.94)	16.72 (1.22)
Estimated verbal IQ (WTAR)	98.66 (3.91)	100.3 (5.17)
Matrix reasoning scaled score (WASI)	8.9 (1.88)	8.38 (2.54)
Handedness – laterality index^a^	87.05 (40.46)	83.83 (39.18)
Years experience taxi driving	13.27 (7.86)	**–**

WTAR = Wechsler Test of Adult Reading; WASI = Wechsler Abbreviated Scale of Intelligence.

a Edinburgh Handedness Inventory.

**Table 2:** Performance of both groups on the New Town tests.

New Town	Taxi drivers Mean (SD)	Control participants Mean (SD)
Learning		
Short film clip recognition (/16)^a^	15.80 (0.52)	15.61 (0.77)
Environmental knowledge		
Scene recognition (/32)^a^	22.3 (2.95)	22.1 (3.19)
Proximity judgements (/10)	7.1 (1.44)	6.5 (1.29)
Route execution (vector distance, where 0 is perfect performance, and a larger score is poorer)**^TD^**	46.25 (31.32)	70.22 (30.30)
Sketch map number of road segments (/16)**^TD^**	9.05 (3.25)	5.44 (2.61)
Sketch map number of road junctions (/8)**^TD^**	4.30 (1.55)	2.61 (1.97)
Sketch map number of landmarks (/28)**^TD^**	12 (4.63)	8.72 (3.35)
Sketch map landmark placement (/84)**^TD^**	27.65 (14.22)	17.83 (10.89)
Ratings		
Sketch map orientation (scale 1–5)**^TD^**	3.55 (0.99)	2.72 (1.01)
Sketch map overall map categorisation (scale 1–6)**^TD^**	4.05 (1.39)	2.66 (1.18)

^**TD**^Taxi drivers significantly better than control participants.

a Includes correct detections and correct rejections.

**Table 3:** Performance of taxi drivers on the London tests.

London (overall)	Taxi drivers Mean (SD)
Learning	
Short film clip recognition (/16)^a^	15.75 (0.55)
Environmental knowledge	
Scene recognition (/32)^a^^,**L**^	27 (2.31)
Proximity judgements (/10)	6.95 (1.35)
Route execution (vector distance, where 0 is perfect performance, and a larger score is poorer)	62.85 (26.91)
Sketch map number of road segments (/25)	8.57 (2.34)
Sketch map number of road junctions (/23)	7.8 (3.69)
Sketch map number of landmarks (/19)^**NT**^	4.47 (2.34)
Sketch map landmark placement (/57)	11.75 (7.95)
Ratings	
Sketch map orientation (scale 1–5)	3.15 (1.08)
Sketch map overall map categorisation (scale 1–6)	3.8 (1.54)
Sketch map integration of existing and new London (scale 1–5)	2.6 (1.14)

**^L^**Significantly better performance for London compared with New Town. ^**NT**^Significantly better performance for New Town compared with London.

a Includes correct detections and correct rejections.

**Table 4:** Performance of taxi drivers on tests of existing and new London – scored separately.

Within London	Taxi drivers Mean (SD)
Existing London	
Scene recognition^a^ – targets (/14)	12.40 (1.27)
Sketch map number of road segments (/14)	9.65 (2.94)
Sketch map number of road junctions (/13)	7.15 (2.03)
Sketch map number of landmarks (/9)	3.35 (2.18)
Sketch map landmark placement (/27)	8.55 (6.67)
New London	
Scene recognition – targets (/10)	6.80 (1.50)
Sketch map number of road segments (/11)	3.75 (2.33)
Sketch map number of road junctions (/10)	3.20 (2.44)
Sketch map number of landmarks (/10)	2.85 (1.63)
Sketch map landmark placement (/30)	6.85 (5.31)
Sketch map orientation (1–5)	1.65 (0.63)
Sketch map overall map categorisation (scale 1–6)	2.0 (1.07)

a Taxi drivers’ mean New Town scene recognition target score was 14.61/24 (SD 3.03). See Results for details of comparisons between the existing London, new London, and New Town.
